# The impact of COVID-19 on the well-being, education and clinical practice of general practice trainees and trainers: a national cross-sectional study

**DOI:** 10.1186/s12909-022-03174-4

**Published:** 2022-02-19

**Authors:** Lotta Coenen, Louise Vanden Poel, Birgitte Schoenmakers, Arne Van Renterghem, Guy Gielis, Roy Remmen, Nele R. Michels

**Affiliations:** 1 Department of Family Medicine and chronic care, Vrije Universiteit Brussels, Laarbeeklaan 103, 1090 Brussels, Belgium; 2 Faculty of Medicine, KU Leuven, ON II Herestraat 49 - box 400, 3000 Leuven, Belgium; 3 Department of Public Health and Primary care, KU Leuven, Kapucijnenvoer 7 blok h - box 7001, 3000 Leuven, Belgium; 4 Department of Public Health and Primary care, Ghent University, Ghent, Belgium; 5 Interuniversity Centre for the Education of General Practitioners, Kapucijnenvoer 33 - Blok H - bus 7001, 3000 Leuven, Belgium; 6 Centre for General Practice, Department of Family Medicine and Population health, Faculty of Medicine and Health Sciences, University of Antwerp, Antwerp, Belgium

**Keywords:** COVID-19, General Practice, Medical education, Workplace learning, Mental health, Health care organization, Telehealth

## Abstract

**Background:**

COVID-19 has changed General Practice (GP) education as well as GP clinical activities. These changes have had an impact on the well-being of medical trainees and the role of GP plays in the society. We have therefore aimed to investigate the impact that COVID-19 has had on GP trainees and trainers in four domains: education, workload, practice organization and the role of GP in society.

Design: a cross-sectional study design was used.

**Methods:**

The Interuniversity Centre for the Education of General Practitioners sent an online survey with close-ended and open-ended questions to all GP trainees and trainers in Flanders, active in the period March – September 2020. Descriptive statistics were performed to analyze the quantitative data and thematic analysis for the qualitative data.

**Results:**

216 (response 25%) GP trainees and 311 (response 26%) trainers participated. GP trainees (63%, *N* = 136) and trainers (76%, *N* = 236) reported new learning opportunities since the COVID-19 pandemic. The introduction of telehealth consulting and changing guidelines required new communication and organizational skills. Most of the GP trainees (75%, *n* = 162) and trainers (71%, *n* = 221) experienced more stress at work and an overload of administrative work. The unfamiliarity with a new infectious disease and the fact that COVID-19 care compromised general GP clinical activities, created insecurity among GP trainers and trainees. Moreover, GP trainees felt that general GP activities were insufficiently covered during the COVID-19 pandemic for their training in GP. GP trainers and trainees experienced mutual support, and secondary support came from other direct colleagues. Measures such as reducing the writing of medical certificates and financial support for administrative and (para) medical support can help to reprioritize the core of GP care.

COVID-19 has enhanced the use of digital learning over peer-to-peer learning and lectures. However, GP trainees and trainers preferred blended learning educational activities.

**Conclusions:**

COVID-19 has created learning opportunities such as telehealth consulting and a flexible organization structure. To ensure quality GP education during the pandemic and beyond, regular GP care should remain the core activity of GP trainees and trainers and a balance between all different learning methods should be found.

**Supplementary Information:**

The online version contains supplementary material available at 10.1186/s12909-022-03174-4.

## Background

Since the first cases of COVID-19 presented itself in China in December 2019, the virus has spread rapidly around the globe [1] Due to the rapid growth of the pandemic in Belgium, the national government decided to enforce a lockdown regime starting from 18th of March 2020 [2]. Universities were closed, and education was converted to Online learning. Similar policies were applied in many countries [3–7]. In this ever-changing world, training doctors may also have become increasingly challenging.

In some countries, trainees’ clinical activity was suspended, whereas in other countries they may have been actively engaged in COVID-19 care [6, 8, 9]. The level of active participation in COVID-19 varied over specialties [8–10]. Studies have suggested that trainees’ education has vastly changed because of COVID-19 [5–11]. Firstly, lectures were converted to online learning [6–8, 12]. Secondly, education opportunities were reduced, [13] given that COVID-19 care was prioritized [6–10]. Moreover, trainees had more responsibilities and a higher workload because of service reorganization [5, 7, 8, 10]. Furthermore, medical professionals have been more prone to infection by COVID-19, which has required additional service organization [14]. Regarding training activities, this often meant a reduction in supervision and intervision sessions [8, 9, 11]. Additionally, research has suggested that working as a medical professional during the COVID-19 pandemic had a major impact on trainees’ well-being, such as an increase in stress, anxiety and loneliness [8–12, 15, 16].

Whereas the altered work-life balance of in-hospital trainees was reported, little attention has been paid to their educators’ experiences. With COVID-19 causing a drastic change in practice organization and as such medical educators had to alter the structure of education activities [7–10]. Formal education activities were limited and instead were switched to more opportunistic educational discussions [8–11, 17]. Furthermore remote clinical supervision has been introduced [7, 8, 10, 11, 17]. In a short period of time peer-to-peer sessions and lectures had to be switched to online sessions. The literature describes study models to facilitate the switch in education organization, however educator’s experience is seldom to be evaluated [17–20]. Furthermore, several studies have described the experiences of in-hospital trainees engaged in COVID-19 care and the consequences for their education [7, 9, 11]. However, only one study investigated the experiences of General Practice trainees (GP trainees) [10]. Therefore, we investigated the impact of COVID-19 on well-being, education and clinical practice of both GP trainees and General Practice trainers (GP trainers). We aimed to identify challenges, opportunities and support systems for GP trainees and GP trainers concerning four domains: education, workload, practice organization and the function of general practice (GP) in society.

### Study context

The GP education in Flanders is provided through an interuniversity program by all four Dutch-speaking medical schools and is coordinated by the Interuniversity Centre for the Education of General Practitioners (ICHO).

Most of the time, GP trainees are active in clinical practice (80%), where they work autonomously with daily supervision of a GP trainer. Besides clinical practice, trainees invest their time in organized study activities such as lectures, research activities and in improving their personal learning curve. Moreover, they participate in seminars composed of 10-12 trainees coached by a GP tutor. During these sessions, GP trainees can discuss and learn from their peers in a safe environment.

The COVID-19 pandemic has affected the organization of the GP trainees’ program in several ways. Since 18th of March 2020, the Belgian government decided to put all non-urgent medical health care on hold. On 27th of April 2020, regular health care was reassumed [2, 21]. GP trainees were engaged in their GP trainers’ practice as well as in COVID-19 specific centers. Besides the organization of COVID-19 care centers, the Belgian health authorities decided to remunerate telephonic and video consults and telehealth consulting was actively promoted and implemented in GPs work [22]. Since 18th of March 2020, all seminars, lectures and exams were converted to online sessions, except for seminars during the summer holidays.

When the Belgian government decided to put all non-urgent medical health care on hold, 5/100,000 inhabitants per day were diagnosed with COVID-19. By May 30th this number decreased to 0.2/100,000. However, when the survey was launched in September 2020 the daily incidence of new cases increased up to 28/100,000 [23, 24].

## Methodology

Data was collected through an online survey. The survey encompassed five sections and aimed to obtain quantitative and qualitative data. Firstly, we surveyed the demographics of the participant and the characteristics of their practice. Secondly, the survey focused on four thematic sections: education, GP practice organization, workload and role of the GP in society during the COVID-19 pandemic. These themes were identified based on the limited existing literature and concerns raised by representatives of GP trainees and trainers. Each section included three closed-ended questions, mainly answered by a 5-point Likert scale, and one or two open-ended questions. Open-ended questions aimed towards creating a better understanding of the quantitative data and to gain more insight in participants’ experiences. The survey was developed by LVP, RR, and GG. To verify face validity and content validity, the questions were reviewed, piloted, and approved by the GP trainers, trainees and medical faculty representatives of the Educational Program Committee (POC). The survey was designed in Qualtrics® and sent to participants in September 2020, using the official communication channels of ICHO in order to reach all Flemish GP trainees (*N* = 852) and GP trainers (*N* = 1204), active in the period March – September 2020. Before completing the online survey, an informed consent was asked from the participants. The survey is available in Additional file 1 (survey for GP trainees) and Additional file 2 (survey for GP trainers).

The data collected by the online survey were analyzed anonymously. Only respondents who answered demographic and at least one closed-ended question were included. Descriptive statistics were used to create an understanding of the quantitative data, by creating frequency Tables. GP trainers were compared to GP trainees using the Chi-squared test. The qualitative dataset was initially analyzed separately by authors LC, GP trainee, and AVR, GP trainer. A thematic analysis was conducted within a realist approach. Semantic themes were identified by using an inductive approach [25, 26]. Secondly, both authors defined and wrote down together the themes that had been found to understand the data to its fullest [26]. Given that one of the open-ended question was formulated in an ambivalent way, it was impossible to know whether respondents’ answers were indicating positive or negative elements. Therefore, it was eliminated from further analysis. Between-method triangulation was performed to create a better understanding of the descriptive statistics and qualitative results and to detect convergent findings [27, 28].

The study design was approved by the Ethical Committee of the Antwerp University Hospital/University of Antwerp, study number 20/51/709.

## Results

The demographics of the participants are represented in Table 1.

**Table 1:** The demographics of the participants both in absolute numbers (N) and in percentages (%)

	GP trainees (*N* = 216)		GP trainers (*N* = 311)	
	N	%	N	%
Gender				
Female	154	71.3	159	51.1
Male	62	28.7	152	48.9
Type of GP practice				
Group practice	118	54.6	185	59.5
Duo practice	33	15.3	60	19.3
Solo practice	28	13.0	47	15.1
Community health center	18	8.3	18	5.8
Hospital	19	8.8	1	0.3
Location of GP practice				
Rural	40	18.5	78	25.1
Suburban	86	39.8	121	38.9
Urban	71	32.9	111	35.7
Amount of previously trained GP trainees				
0 trainees			24	7.7
1 - 3 trainees			148	47.6
4 - 10 trainees			94	30.2
> 10 trainees			45	14.5

Twenty-five percent (*N* = 216) of GP trainees completed the survey, of which 71% were female. Half of the GP trainees (55%) were working in a group practice (> 2 graduated GPs), others were active in a duo practice (15%), solo practice (13%), community health center (8%) and hospital setting (9%).

Twenty-six percent (*N* = 311) of GP trainers participated, of which 51% were female. GP trainers were active in a group practice (59%), duo practice (19%) or community health center (6%). Only 15% had a solo practice. Half of the GP trainers (48%) had minor experience as a trainer and had previously trained 1 – 3 trainees, 8% had no prior experience. Whereas 15% of the GP trainers had supervised more than 10 trainees, 30% had moderate experiences by guiding 4 – 10 trainees in the past. In the following paragraphs, for each section we report the quantitative data, presented in Table 2, followed by a qualitative analysis of the qualitative information.

**Table 2:** Representation of the quantitative data

	GP trainers		GP trainees		Degrees of freedom	Sample size N	*X* ^2^ statistic value	*p* value
	Mean	95% CI	Mean	95% CI				
**Education**								
Opportunity to learn new aspects of GP	3.85	3.74 – 3.97	3.39	3.23 – 3.55	4	527	27.5	< 0.001
Quality of learning opportunities^a^	1.60	1.54 – 1.67	1.29	1.22 – 1.36	2	527	46.5	< 0.001
Appreciation of online lectures			3.05	2.89 – 3.20				
Quality of online seminars			1.43	1.35 – 1.50				
Accessibility of GP coach			2.93	2.83 – 3.02				
**Practice organization**								
Improving risk management	3.44	3.33 – 3.56	3.36	3.24 – 3.48	4	493	11.2	0.03
How to perform telehealth consultations	4.51	4.43 – 4.60	4.24	4.12 – 4.37	4	493	17.3	0.002
Work in out-practice COVID-19 care centers	3.79	3.63 – 3.95	4.05	3.87 – 4.24	4	493	5.2	0.27
**Workload**								
Working hours^a^	1.98	1.90 – 2.06	2.26	2.15 – 2.36	2	485	15.9	< 0.001
Stress at work^a^	2.07	2.01 – 2.13	2.16	2.09 – 2.24	2	485	3.8	0.15
Intensity of on-call shifts	3.46	3.33 – 3.59	4.04	3.89 – 4.18	4	485	33	< 0.001
**The role of GP in society**								
Cooperation with other health care providers	3.33	3.25 – 3.42	2.87	2.77 – 2.96	4	476	36.5	< 0.001
Support by the government	2.18	2.05 – 2.31	1.97	1.82 – 2.11	4	476	9.3	0.05

Means and confidence intervals according to a 5-point Likert scale varying from strongly disagree (1) to strongly agree (5)) as well as the comparison of GP trainers to GP trainees. ^a^ 3-point Likert scale

### Education

#### Descriptive statistics

**Fig. 1:**
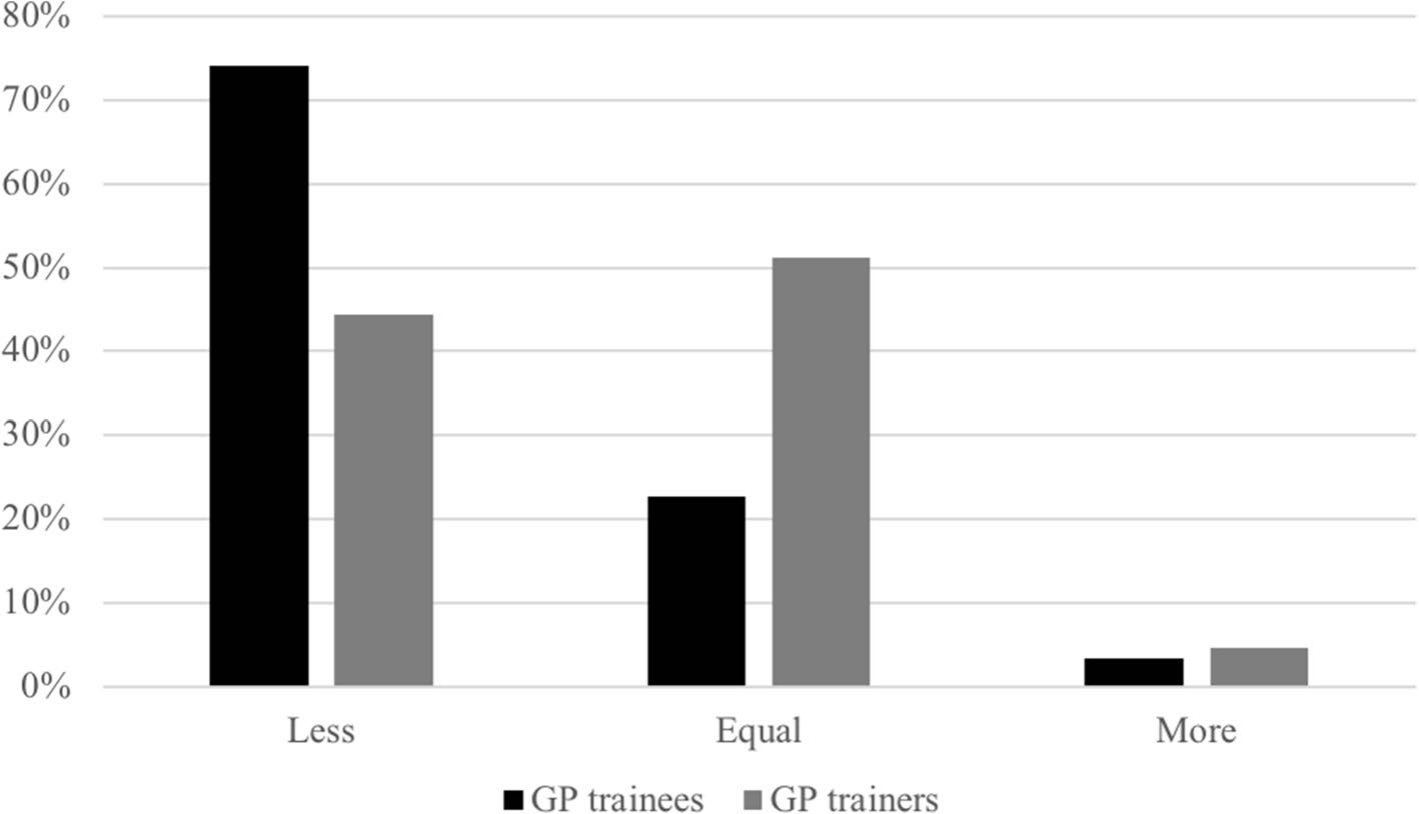
Quality education moments during the COVID-19 pandemic compared to the period before. Percentage of answers on the question ‘if quality education moments were facilitated less, equal or more often during COVID-19 pandemic compared to the period before’

A vast majority of both GP trainees (63%) and GP trainers (76%) believed that COVID-19 had offered new and different learning opportunities. Half of the GP trainers (51%) indicated that the quality of learning opportunities in clinical practice during the COVID-19 pandemic was equal to the period before the pandemic, whereas only 23% of the GP trainees agreed. This contrast is visualized in Fig. 1 and Table 2. Up to 74% of the GP trainees believed that the quality of these learning opportunities had decreased. GP trainees were divided in their appreciation of online lectures as presented in Table 2: 33% experienced online lectures as effective but another 39% disagreed and 28% neither agreed nor disagreed. Almost all of the seminars (94%) were switched to online sessions. Three out of five GP trainees (59%) experienced the online seminars to be of lower quality, 39% reported that the quality was equal. Only 2% thought the quality of the seminars had increased during the COVID-19 pandemic. However, 74% of the GP trainees were able to approach their GP coach as easily as before.

#### Thematic analysis

According to the open-ended questions presented in Table 3, GP trainees and trainers acquired similar skills during the COVID-19 pandemic. Main topics learned by both groups were executing telehealth consulting, improving knowledge on the COVID-19 virus, pathology and flexibility due to the ever-changing working circumstances and guidelines during the pandemic. Moreover, GP trainees were able to improve their organizational skills whilst GP trainers were more likely to appreciate a well-organized cabinet and collaboration with other health care partners such as GP colleagues, hospitals, and primary care networks. GP trainees have learned to improve communication skills towards patients and colleagues in many aspects. Clear communication with colleagues and patients was perceived as an important factor in the flexibility and organization of the working circumstances. New communication skills were especially obtained by telehealth consulting and providing care in the absence of physical contact.

**Table 3:** The thematic analysis of learning opportunities as experienced by GP trainers and trainees

Education opportunities			
	Themes	Specifications	Citations
GP trainers and trainees	Flexibility	Implementing ever-changing guidelines, crisis management	
	Practice organisation and management	Teamwork, appointments, practice organisation, population health	GP trainee: *“They expected I helped in practice organization, experience was less important during the pandemic.”*
	Knowledge on COVID-19		
	Telehealth consults	New communication channels, detecting red flags	
GP trainers	Cooperation	Other GP practices, COVID-19 testing, hospitals	
	Online lectures	No traveling time needed	
	GP trainees	Better knowledge of new guidelines	*“GP trainees are real doctors, they easily get used to and implement to new guidelines and corresponding practice organisation.”*
GP trainees	Communication skills	Patient education, bad news, anxiety	
	Self care	Personal limits, infection prevention	

However, despite several new learning opportunities, GP trainees indicated an even greater loss in learning possibilities related to the regular GP practice and care. The full scope of challenges is shown in Table 4. Online lectures as well as seminars were found to be of lower quality, due to the obliged online format but also due to the predominance of COVID-19 over other essential learning objectives. Especially peer-to-peer learning was limited due to a lack in face-to-face interactions. GP trainers appreciated their online sessions because no traveling time was needed.

**Table 4:** The thematic analysis of education challenges as experienced by GP trainers and trainees

Education challenges			
	Themes	Specifications	Citations
GP trainers and trainees	Lack of regular GP care	Lack of chronic care, health prevention, care postponed, monotonous	GP trainer: *“I admit my GP trainee was responsible for COVID-19 care so I could focus on regular health care for my patients.”*
	Telehealth consults	Lack of in-person contact with patients, depth, time consuming	
	Administrative tasks	Sick leave notes, ever-changing guidelines, IT troubles	GP trainee and trainer: *“I feel more like a secretary than a doctor.”*
GP trainers	Self care	Difficulties in work-life balance	
GP trainees	Patients	Impatient, less understanding, patients are scared of coming to the cabinet	
	Less intervision	With GP trainer and other direct colleagues, lower quality of seminars	
	Lectures		*“Lectures seem of less importance than before COVID-19 and the quality is lower.”*

### Practice organization

#### Descriptive statistics

Practice organizations have had to adapt to the health crisis in several ways. COVID-19 has changed the perception of good risk management by 49% of GP trainees and 56% of the trainers. For the first time telehealth consults were financially remunerated in Belgium and were introduced as such in the daily life of GP [22]. Most of GP trainers (93%) and GP trainees (88%) indicated that they learned how to perform these telehealth consultations. Eight out of ten (79%) GP trainees indicated to work in out-practice COVID-19 care centers, but only 71% of GP trainers confirmed that their trainee engaged in such services, see Table 2.

#### Thematic analysis

Table 5 shows that COVID-19 challenged practice organization in many ways.

**Table 5:** GP trainers and trainees perceived a negative impact of COVID-19 in practice organization and in their personal lives

Negative impact of COVID-19			
	Themes	Specifications	Citations
GP trainers and trainees	Telehealth consults	Intense, new way of consulting, overwhelming regular consultations	*“call your GP-phenomene”*
	Ever-changing guidelines		*“Guidelines adapting continuously to evolving pandemic requiring practice re-organisation at multiple times.”*
	Government		*“GP care was at the center of COVID-19 care, however we were rarely consulted in the policy-making. Therefore, the character of our profession changed profoundly.”*
	Administrative tasks	Sick leave notes, implementing test strategies, information for patients, contact tracing	
	Increasing workload	COVID-19 care, regular health care, catching up postponed health care	
	Insecurity and anxiety	Unknown disease, fear to misdiagnose, lack of personal protection material, scared of infecting family or friends	
	Lack of passion for the job		
	New content of GP practice		*“COVID-19 care creates monotonous work which changed the character of GP care profoundly.”*

GP trainers indicated that a good practice organization, including administrative support, strict working hours and appointments, has helped to tackle the COVID-19 pandemic. They perceived the lack of governmental support and the lack of regular GP care as main organizational challenges in addition to finding a right balance between regular and COVID-19 care. GP trainers admitted to being tempted to engage the GP trainee mainly or only for COVID-19 care. Changes to the governmental- and scientific guidelines were insufficiently communicated to the population, as well as a lack of personal protection material were experienced. GP trainees and trainers experienced long phone calls from patients and overwhelming administration work, including writing sick-leave certificates and testing on COVID-19 for non-medical purposes.

Additionally, both GP trainees and trainers indicated that telehealth consulting was a major challenge. They often felt that physical contact was missing and as a result compromised patient’s understanding. A fear of missing important diagnoses besides COVID-19 was reported. GP trainees also experienced more difficulties reaching out to vulnerable patient groups because of the new ways of consulting. All those elements were perceived as a strain on the work- (private) life balance according to GP trainers.

### Workload

#### Descriptive statistics

**Fig. 2:**
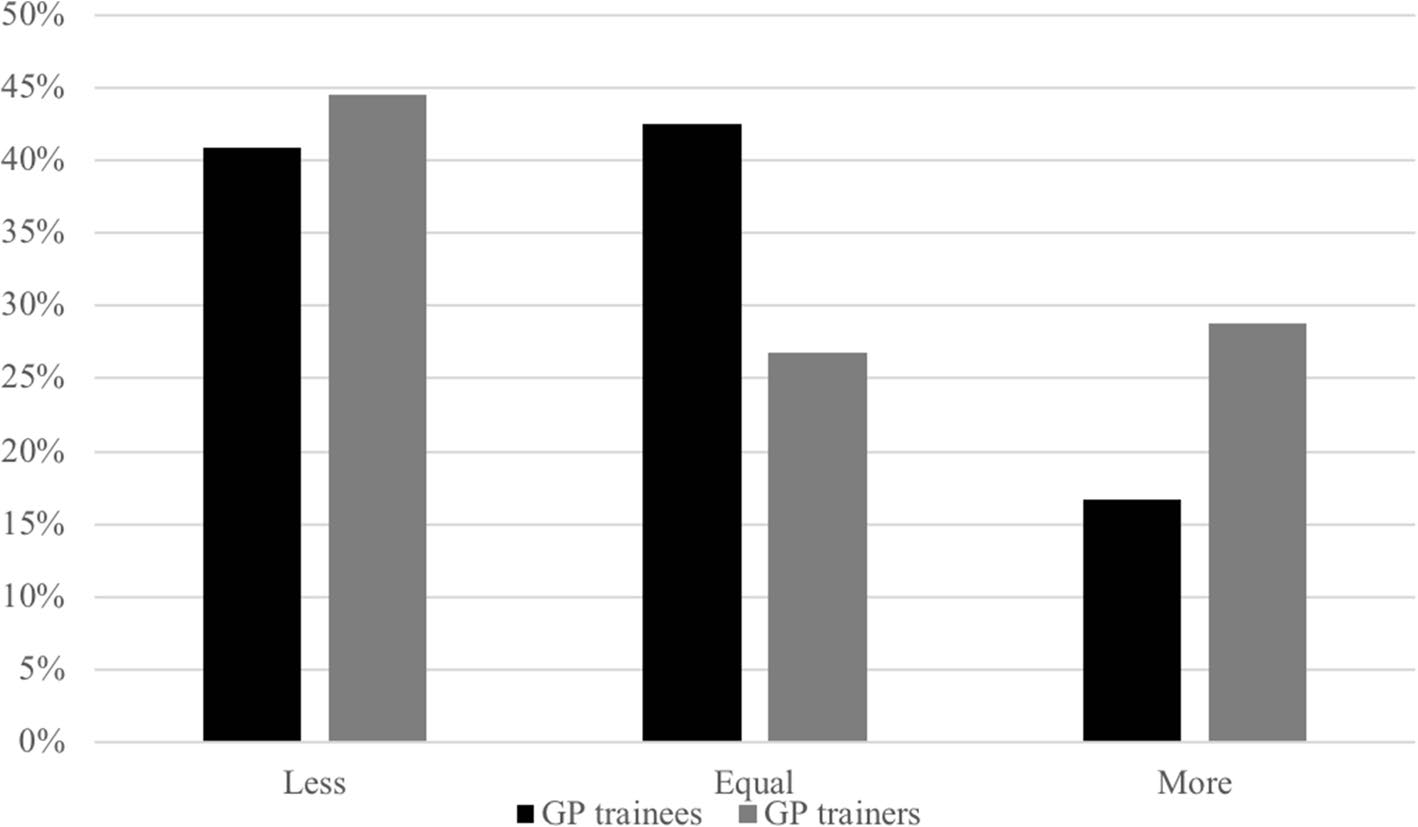
Working hours during the COVID-19 pandemic compared to the period before. Percentage of answers on the question ‘if GP trainees worked less, equal or more hours during the COVID-19 pandemic compared to the period before’

Working hours were very diverse amongst the respondents. As is shown in Fig. 2 and Tables 2, 42% of the trainees worked more during the COVID-19 pandemic, 17% worked less and 40% indicated that their working hours did not change. However, 29% of GP trainers reported that their trainee worked less hours. GP trainees experienced more stress at work since COVID-19 (71%) and the on-call shifts as far more intense (72%). GP trainers (75%) also experienced more stress at work but only half of them (52%) thought on-call shifts were more intense for their trainee.

#### Thematic analysis

Overall, respondents regularly indicated that the workload during the first period of higher infection rates (March-May 2020) was acceptable, whereas the workload during the beginning of the second period of increasing infection rates (September 2020) was intolerable and is still increasing. When non-urgent care resumed, a balance between all different tasks had to be found. However, COVID-19 remained an overall priority and especially GP trainees felt that their work was still monotonous and concentrated on the pandemic. The increased workload and work stress were mainly caused by the changing guidelines and health care organization requirements, of which GP played a main role. COVID-19 pushed back the core practice of GPs, posing a major organizational and motivational challenge. The administrative overload and communication with patients were experienced as a main strain. Mainly GP trainees experienced patients as less tolerant and understanding, especially over the phone. GP trainees and trainers reported an important fear for the virus itself, including a lack of knowledge on the COVID-19 pathology. However, the main fear of the respondents was getting infected with COVID-19 themselves or to infect their close contacts. In addition, some respondents indicated to avoid personal contact with their peers, despite their emotional needs.

Motivating factors were very similar for GP trainees and trainers, as can be seen in Table 6. Overall, they experienced most support from their direct colleagues and peers in formal and mostly informal discussions. GP trainers indicated their trainee was of great support in keeping up to date with COVID-19 guidelines. An important sense of solidarity amongst GPs from different practices was established during the pandemic. A good organization of working shifts and efforts of administrative or nursing employees could decrease the workload. Outside the work setting the respondents found distraction and motivation by planning free time and spending time with their friends and family. Many of them continued or increased sport activities and meditation.

**Table 6:** The support systems experienced by GP trainers and trainees during the COVID-19 pandemic

Support systems			
	Themes	Specifications	Citations
GP trainers and trainees	Colleagues	Venting, solidarity	
	Practice organization	Structured work, clear working hours, administrative support	
	Personal network	Family, partner, friends	
	Perspective		*“At least we have a job.”* *“It is only temporary.”*
	Clear guidelines	COVID-19 guidelines regarding testing, case identification	
	Time off	Vacation trips, leisure time activities, sports, meditation	
GP trainers	Patient’s support	Personal protection material, recognition	

### The role of GP in society

#### Descriptive statistics

Since the beginning of the pandemic, GP trainers (48%) reported an increase in their cooperation with other health care providers compared to only 19% of GP trainees, see Table 2. In terms of support by the government, 74% of GP trainees and 69% of GP trainers felt insufficiently supported.

#### Thematic analysis

By end of September 2020, the respondents noted an increase in the numbers of patients infected with COVID-19 and many patients were less obedient to the government restrictions imposed to limit the spread of COVID-19. Table 7 shows the measures the respondents propose to improve COVID-19 care and GP care in general.

**Table 7:** The thematic analysis of proposed measures to improve GP care during the COVID-19 pandemic and beyond

Measures to improve GP care during the COVID-19 pandemic and beyond			
	Themes	Specifications	Citations
GP trainers and trainees	Administrative simplification	Uniform sick leave notes, patient education, IT tools, improving telehealth	
	Financial support	Telehealth consults, administrative employees, nursing employees, remuneration for multidisciplinary practices	
	Recognition for GP care	Participations of GP representatives in policy making, recognition for the central role of GPs in the health care provision to all	
	Communication	Clear guidelines and regulations towards the population, communication to subgroups	
	Patient education		*“Patients should be able to take more responsibilities to improve their health and understand possible symptoms.”*
	Crisisplan	Better preparation for possible future health crisises by policy makers	
GP trainers	Increasing cooperation	With policy makers, other health care providers, paramedics	
	Health prevention	Vaccinations, preventive health care, information for the population	

GPs asked for recognition of the role of primary care and GP in tackling the COVID-19 pandemic as well as in general health care. Therefore, GP trainees as well as trainers asked for more involvement in decision making processes concerning their work, finances, and organization. For GP trainees and trainers, the need to simplify administrative issues regarding sick-leave certificates was a major issue of concern.

Respondents had been asking for financial support, in order to make it possible to develop multidisciplinary practices. GP trainers also pleaded for remuneration for telehealth consulting to be permanent. GP trainees and trainers stated that they would like better cooperation amongst primary care networks as well as other health care workers. IT solutions and e-health platforms can play an interesting role in improving cooperation according to several GP trainers, whereas GP trainees more often indicated the need for nurses or paramedics in performing COVID-19 testing, especially for asymptomatic people.

However, improvements are not only required within GP practice; respondents also saw a need to focus on patient education and empowerment. Moreover, they claimed the need for an action plan in preparation for a future health crisis.

Both GP trainers and trainees felt an important change in clinical practice and a decrease in actual health care and prevention care since March 2020. Thence and the COVID-19 pandemic, 24 (11%) GP trainees and 13 (4%) GP trainers spontaneously mentioned losing their passion for their profession and one respondent actively considered quitting as a GP.

## Discussion

We aimed to investigate how GP trainees and trainers experienced the changes in education and clinical practice during the COVID-19 pandemic. This study showed that the COVID-19 pandemic created challenges as well as opportunities for GP trainees and trainers in their clinical practice and during the educational activities. Interestingly, challenges were often interlined to the opportunities. Hereafter, we will discuss how these challenges interact with the opportunities.

### Education

Both groups of participants experienced new learning opportunities due to the COVID-19 pandemic. Mostly, organization skills were developed due to the changing guidelines for COVID-19 treatment and testing strategy [12]. In addition, the introduction and implementation of telehealth consulting required new communication skills [29–31]. GP trainees and GP trainers had to learn and recognize red flags, to diagnose and to explain treatments without any physical examination. GP trainers were challenged by learning new skills and teaching and supervising these competences in the very same moment [8, 17, 18]. The implementation of the COVID-19 guidelines and the organization of separate consultations for infectious diseases required flexibility and also the need to quickly adapting. GP trainees were actively engaged in the implementation of the guidelines and therefore acquired new organizational skills [17].

GP trainees noticed that online lectures and seminars were missing impact in comparison to in-person sessions [18, 19, 32]. Moreover, seminars were experienced as an important moment to vent and exchange about the impact of COVID-19. GP trainees felt that virtual sessions did not allow venting and exchanging experiences as much as the former in-person sessions did. Currently, lectures and seminars have been integrally transferred into online formats. However, recent literature showed that an in-person session cannot simply be switched to an online session [7, 8, 33]. Several alternative online learning tools should be integrated, especially in the case of peer-to-peer sessions [7, 9, 10, 33, 34]. Our study confirmed that online learning can provide opportunities thanks to the ‘anywhere/anytime’ principle, but we noticed the advantages and continued appreciation of students towards in-person peer-to-peer learning. Blended learning would be preferable [7, 9, 10, 33, 34].

### Practice organization

GP trainees experienced that COVID-19 was overrepresented in their clinical work. Moreover, GP trainers admitted they were tempted to direct COVID-19 cases more often to their trainee. Based on the qualitative data, we assume that GP trainers maintain the professional relationships with their patients, by continuing to focus on chronic healthcare. Moreover, GP trainers assume their trainee is more familiar with the COVID-19 guidelines. As such, the GP trainees lacked learning opportunities in common GP clinical work. Studies amongst different medical specialties confirmed that residents are lacking general clinical practice during the COVID-19 pandemic, as regular care have been postponed [5, 9–11, 32, 35]. Residents were very willing to assist in (primary) medical care during the pandemic but feared an inferior education for their future medical profession [6–8, 10].

### Workload

GP trainees and trainers were overwhelmed by the amount of administrative work and experienced more stress since the COVID-19 pandemic. Telehealth consulting reduced actual clinical consults but have not been able to replace them [30, 36]. Throughout telehealth consulting, GP trainees as well as their trainers feared missing important diagnoses [29, 36]. Moreover, GP trainees indicated that a lack of common history with the patients increased their insecurity. Other studies confirmed that telehealth consults increased insecurity amongst residents [8–10, 29, 30, 36, 37]. Eventually, the selection of cases suitable for telehealth consulting should be reviewed and more targeted to follow-up consults instead of acute symptomatology such as respiratory infections [10, 29, 30, 36–38].

Seventy-two percent of GP trainees experienced on-call shifts as more intense since the COVID-19 pandemic, but this was recognized by only 52% of GP trainers. Literature confirmed that on-call shifts were experienced to be more intense [9–11]. No clarification for this major difference in perception between GP trainees and their trainers could be found. We presumed that the lack of formal supervision could diminish the ability to discuss out-of-practice activities [10, 31]. However, tele supervision does not imply any safety risks for patient’s health care and therefore should not increase insecurity amongst GP trainees [10, 17, 20, 31]. During on call shifts, patients are less familiar to the GP trainee, and this might increase the distress for misdiagnosis [30]. Furthermore, the organization of on call shifts changed regularly because of the evolving COVID-19 pandemic and changing guidelines. Whereas GP trainers can rely on their experience in these unpredictable circumstances, GP trainees cannot [30, 36].

### The role of GP in society

GP trainers and trainees felt that COVID-19 was taking over regular GP work and has impacted their overall work satisfaction. Although we did not survey job appreciation systematically amongst our respondents, one out of 10 GP trainees and one out of 25 GP trainers spontaneously mentioned that they have lost their job satisfaction. Other studies outlined increasing mental health issues during clerkships and amongst trainees, but to our knowledge no other study has previously examined this with GP trainees [8–11, 34]. Considering these results, we feared GP trainers and trainees might eventually quit their medical career.

Measures such as reducing sick-leave notes or other medical certificates and financial aid for administrative and (para-)medical support, need to be put in place in short and long term to improve the work life and work content of Belgian GPs. Moreover, there is a need to increase financial and moral recognition for the role GPs have in the health care system, in society and in their patients’ lives to safeguard the core of GP practice [9, 11, 35].

### Strengths and limitations

By including both GP trainers and trainees, the perspective of all main actors in GP education was investigated in this study. Only one other study was found with a similar set-up, [10] in most other studies, only the trainees were interviewed [8, 10]. As GP education in many countries includes workplace learning, the role of the GP trainer is of major importance [5, 10, 20]. By including their perspective and comparing to GP trainees, the challenges and possibilities for the organization of education during a pandemic could be fully described.

The overall response rate of 26% is a reasonable response rate for online surveys and sufficient for a confidence level of at least 80% [39, 40]. Although we used the official communication channels for GP trainers and GP trainees, the lower response rate may be due to an increasing workload because of the rising COVID-19 infections and the insufficient use of boosting methods to encourage GP trainers and trainees to participate [40, 41]. Moreover, the response rate for online surveys is known to be 10% lower than for other survey methods despite the use of encouraging methods such as reminders or incentives [40, 42]. However, both groups of respondents, GP trainers and trainees, were representative for gender and region of practice compared to all GP trainers and trainees in Flanders. Whereas other studies focused on a specific university or local region only, this survey was sent out to all GP trainers and their trainees in Flanders [10, 34].

As the COVID-19 pandemic developed rapidly, a swift response to the impact on GP trainees and trainers was essential for the educational organization. Therefore, only content and face validity were assessed in the study design. It can be seen as a limitation that reliability or construct validity were not measured. However, this study combined descriptive statistics and qualitative thematic analysis of responses to open-ended questions to better understand the impact on GP education during the COVID-19 pandemic. The thematic analysis created the opportunity to further clarify and find in-depth information on the quantitative findings.

### Future research

The COVID-19 pandemic stresses the processes and outcomes of GP education. Therefore, a profound investigation of threats for job satisfaction and the support systems of our trainees and trainers should be on the agenda in order to cope with future pandemic threats and to facilitate the GP core clinical activities. Online learning could be further developed and evaluated and fully integrated in blended workplace learning. Telehealth consults should be actively included in the education program for GP trainers and trainees.

## Conclusion

COVID-19 has had a major impact on the education and day-to-day work of GP trainees and GP trainers. With online learning being introduced, GP trainers and trainees reported advantages and disadvantages to Online learning, as such blended learning would be preferable.

New skills were acquired during the COVID-19 pandemic such as telehealth consults, communication and organizational skills. However, GP trainees and trainers experienced multiple challenges because of COVID-19: an administrative overload, a mandatory focus on COVID-19 care and increased insecurity in diagnoses as well as personal protection. GP trainees especially lacked peer-to-peer contact. GP trainers’ and trainees’ job satisfaction decreased because of the current working circumstances and work content.

Good practice organization, collaboration and communication with colleagues were essential to face the challenges posed by COVID-19. Other proposed measures to regain the core of GP care were administrative simplification and financial support for administrative and (para) medical support in GP cabinets.

Further qualitative and quantitative research may help to further identify and understand the challenges and support systems for GP and GP workplace-based learning in times of stress on the health system like the COVID-19 pandemic. Additionally, the measures to improve the position of GP in the health system should be placed on decision makers’ agenda.

## Supplementary Information

**Additional file 1.** Study survey for GP trainees.

**Additional file 2.** Survey for GP trainers.

## Data Availability

The datasets used and/or analysed during the current study are available from the corresponding author on reasonable request.

## Abbreviations

GP: General Practice
ICHO: Interuniversity Centre for the Education of General Practitioners
POC: Educational Program Committee
