# Supplementary material for: The impact of COVID-19 on the well-being, education and clinical practice of general practice trainees and trainers: a national cross-sectional study
Source: BMC Med Educ. 2022 Feb 19;22:108. doi: 10.1186/s12909-022-03174-4 (PMC8857395; doi:10.1186/s12909-022-03174-4)
Supplement: Supplementary file 1 — Additional file 1. Study survey for GP trainees. [file 12909_2022_3174_MOESM1_ESM.docx]

**APPENDIX 1: Study survey for GP trainees**

**Demographics**

1. I am …

*(multiple choice, 1 option)*

- - GP trainee
  - GP trainer

1. Gender

*(multiple choice, 1 option)*

- Male
- Female

1. Region of the practice confirm the educational regions

*(multiple choice, 1 option)*

- Aalst-Oudenaarde
- *… (all official regions as described by ICHO)*
- Sint-Niklaas-Dendermonde

1. I am in grade

*(multiple choice, 1 option)*

- - 1
  - 2
  - 3

1. My type of practice (March – September 2020)

*(multiple choice, 1 option)*

- - Solo practice
  - Duo practice
  - Group practice
  - Community health center
  - Hospital

1. Practice location

*(multiple choice, 1 option)*

- Urban
- Suburbs
- Rural

**Education**

Look back to the period March – September 2020 and compare the situation to the situation before that period.

1. This period was an opportunity to learn new aspects of General Practice.

*(5-point Likert scale ranging from Strongly Disagree (1) to Strongly agree (5))*

1. I enjoyed … quality education moments in GP practice during as before the COVID-19 pandemic.

*(multiple choice, 1 option)*

- More
- Equal
- Less

1. How were the seminars organized during the COVID-19 pandemic?

*(multiple choice, 1 option)*

- Mostly online
- A balance combination of online and face to face sessions
- Mostly face to face

1. The quality of the seminars was … to the seminars before the COVID-19 pandemic.

*(multiple choice, 1 option)*

- More
- Equal
- Less

1. The GP tutor was … accessible than before the COVID-19 pandemic.

*(multiple choice, 1 option)*

- Much less
- Less
- Equal
- More
- A lot more

1. I experienced the online lectures as effective.

*(5-point Likert scale ranging from Strongly Disagree (1) to Strongly agree (5))*

1. What have you learned most during the COVID-19 pandemic?

*(Open-ended question)*

1. Which obstacles have you experienced most during the COVID-19 pandemic?

*(Open-ended question)*

**Practice organization**

Look back to the period March – September 2020 and compare the situation to the situation before that period.

1. I learned to reflect different on the patient population of my practice regarding risk factors (e.g. COPD, overweight, poverty, …)

*(5-point Likert scale ranging from Strongly Disagree (1) to Strongly agree (5))*

1. I learned to perform tele consults.

*(5-point Likert scale ranging from Strongly Disagree (1) to Strongly agree (5))*

1. I was involved in out of practice COVID-19 care (such as sampling, tracing, testing, special hospital activities, etc.)

*(5-point Likert scale ranging from Strongly Disagree (1) to Strongly agree (5))*

1. (This question was removed from the analysis) Which organizational developments evolved for the better or the worse during the COVID-19 pandemic?

*(Open-ended question)*

**Workload**

Look back to the period March – September 2020 and compare the situation to the situation before that period.

1. The on call shifts were more intense.

*(5-point Likert scale ranging from Strongly Disagree (1) to Strongly agree (5))*

1. I worked … than before the COVID-19 pandemic.

*(multiple choice, 1 option)*

- More
- Equal
- Less

1. The stress at work was … than before the COVID-19 pandemic.

*(multiple choice, 1 option)*

- More
- Equal
- Less

1. What did help you most to handle the stress at work during the COVID-19 pandemic?

*(Open-ended question)*

1. What were the most stressful factors during the COVID-19 pandemic?

*(Open-ended question)*

**GP in society**

Look back to the period March – September 2020 and compare the situation to the situation before that period.

1. I did collaborate more with the employees/management of residential care centers for the elderly.

*(5-point Likert scale ranging from Strongly Disagree (1) to Strongly agree (5))*

1. I did collaborate more with the employees/management of hospitals.

*(5-point Likert scale ranging from Strongly Disagree (1) to Strongly agree (5))*

1. I did collaborate more with other health care actors such as social workers, psychotherapists, health insurance companies, etc.

*(5-point Likert scale ranging from Strongly Disagree (1) to Strongly agree (5))*

1. The government has supported me and other general practitioners sufficiently during the COVID-19 pandemic.

*(5-point Likert scale ranging from Strongly Disagree (1) to Strongly agree (5))*

1. Which changes are needed the most in the next months and years in GP and health care in general?

*(Open-ended question)*

1. Did you experience a difference over the months the COVID-19 pandemic manifested? What was the reason for these differences?

*(Open-ended question)*
